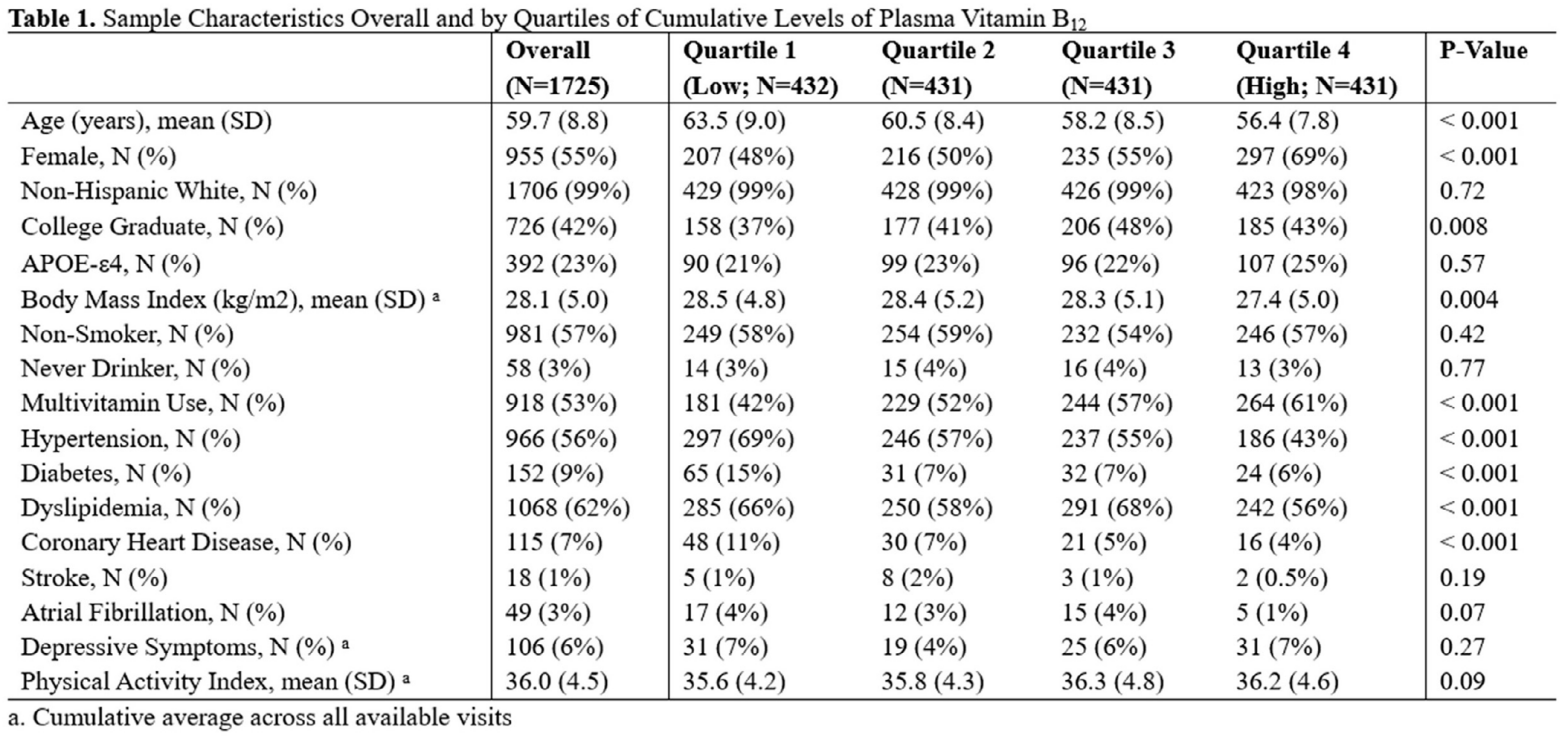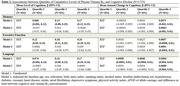# Vitamin B12 and cognitive decline among older adults in the Framingham Heart Study

**DOI:** 10.1002/alz70860_096276

**Published:** 2025-12-23

**Authors:** Francesca R Marino, Rhoda Au, Gail Rogers, Josh W Miller, Jacob Selhub, Paul F Jacques, Phillip H Hwang

**Affiliations:** ^1^ Boston University School of Medicine, Boston, MA, USA; ^2^ Boston University School of Public Health, Boston, MA, USA; ^3^ Framingham Heart Study, Boston University School of Medicine, Boston, MA, USA; ^4^ Rutgers University, New Brunswick, NJ, USA; ^5^ Tufts University, Boston, MA, USA

## Abstract

**Background:**

There is mixed evidence as to whether higher vitamin B_12_ status is associated with better cognitive function. Prior studies are limited by use of self‐reported questionnaires, having only a single measure of B_12_, or having short follow‐up. As such, there is a need for evidence to evaluate whether inadequate B_12_ status measured at multiple timepoints is associated with prospective cognitive decline over longer time periods.

**Method:**

This study leveraged data from 1725 participants in the Framingham Heart Study Offspring Cohort using a combined measure of B12 status, 3cB_12_, that included in one formula information from three biomarkers: plasma cobalamin, homocysteine and methylmalonic acid. Participants were free of dementia at baseline, had ≥2 measures of 3cB_12_, and ≥2 measures of neuropsychological (NP) factor scores. The cumulative average level of 3cB_12_ was calculated across all exams, and then participants were divided into quartiles. NP factor scores for memory, executive function, and language were previously estimated using bi‐factor confirmatory factor analysis models. Separate linear mixed effects models, adjusted for demographic, anthropometric, lifestyle, and medical covariates, estimated the annual change in each factor score comparing participants across quartiles of cumulative average 3cB_12_ scores.

**Result:**

On average, participants were aged 60 years at baseline and 55% female. Those in the highest 3cB_12_ quartile were younger, more likely to be female, had higher educational attainment, and lower prevalence of certain comorbidities than the lowest quartile (Table 1). Over a mean of 17.7 years, participants in the highest 3cB_12_ quartile had attenuated memory, executive function, and language decline compared to the lowest quartile (memory: β: 0.0063, 95% CI: 0.001‐0.01; executive function: β: 0.0051, 95% CI: 0.002‐0.001; language: β: 0.0089, 95% CI: 0.004‐0.01) (Table 2). There were no cross‐sectional associations between 3cB_12_ and any cognitive domain (Table 2).

**Conclusion:**

Higher cumulative average B_12_ status from middle‐ to later‐life is associated with small but significant attenuation in cognitive decline across multiple domains. Efforts to improve nutrition throughout the life course may help mitigate cognitive decline into older age. Future work should evaluate whether targeting nutrition in combination with other modifiable risk factors can better slow cognitive decline.